# A non-ideal approach to slurs

**DOI:** 10.1007/s11229-023-04315-y

**Published:** 2023-09-09

**Authors:** Deborah Mühlebach

**Affiliations:** Freie Universität Berlin, Institut für Philosophie, Habelschwerdter Allee 30, 14195 Berlin, Germany

**Keywords:** Derogatory language use, Semantics, Pejoratives, Swearwords, Derogatory terms, Inferentialism

## Abstract

Philosophers of language are increasingly engaging with derogatory terms or slurs. Only few theorists take such language as a starting point for addressing puzzles in philosophy of language with little connection to our real-world problems. This paper aims to show that the political nature of derogatory language use calls for non-ideal theorising as we find it in the work of feminist and critical race scholars. Most contemporary theories of slurs, so I argue, fall short on some desiderata associated with a non-ideal approach. They neglect crucial linguistic or political aspects of morally and politically significant meaning. I argue that a two-stage project is necessary to understand the perniciousness of slurs: accounting for the derogatory content of derogatory terms in general and, additionally, explaining the communicative function of slurs more specifically. I end by showing how inferentialism is well-suited to account for the content of derogatory terms whilst allowing for further explanations of the communicative functions of slurs.

I don’t know what most white people in this country feel, but I can only conclude what they feel from the state of their institutions. I don’t know if white Christians hate Negroes or not, but I know that we have a Christian church which is white and a Christian church which is black. […] I don’t know whether the labour unions and their bosses really hate me, that doesn’t matter, but I know that I’m not in their unions. I don’t know if the real estate lobbies are against Black people, but I know that the real estate lobbies keep me in the ghetto. I don’t know if the board of education hates Black people, but I know the textbooks that they give my children to read and the schools that they have to go to.

James Baldwin on the Dick Cavett Show 1968

## Introduction

Philosophers of language are increasingly engaging with issues concerning politically and morally significant language. For instance, there is a growing body of literature on the meaning of slurring or derogatory terms. Only few theorists take such terms as a starting point for addressing puzzles in philosophy of language with little connection to our real-world problems. As Geoff Nunberg (2018) observes, most theorists who work on derogatory or slurring terms are motivated by topical political discussions and hope to contribute to a better understanding of the issues involved in these debates.

Better understanding the complex and messy issues involved in our topical political debates about language use calls for non-ideal theorising. Feminist and critical race philosophers who engage in non-ideal theorising usually do so because they try to do justice to our complex and messy social reality. By drawing on recent work on non-ideal philosophy of language (Beaver & Stanley, 2019; Keiser, 2022; Mühlebach, 2022a; Engelhardt & Moran, forthcoming), I aim to show that most contemporary theories of slurs fall short on some desiderata associated with a non-ideal approach to politically and morally significant linguistic meaning. They tend to neglect important linguistic or moral and political aspects of this phenomenon. In order to do justice to all these aspects, I suggest to adopt a taxonomy of swearing, pejorative, derogatory, and slurring terms that is guided by their moral and political significance. According to this taxonomy, most available theories account for the communicative function of slurs, but not for meaning in derogatory and slurring language use that is embedded in oppressive social structures. I argue that a two-stage project is necessary to understand meaning in morally and politically problematic uses of language: accounting for the derogatory content of derogatory terms in general and, additionally, explaining the communicative function of slurs more specifically.

This article proceeds in four stages. Firstly, I sketch the main approaches to derogatory or slurring terms by grouping them into four categories: content views, hybrid views, misrepresentation views, and linguistically minimal views. Secondly, I introduce the idea of non-ideal theorising in philosophy of language. By looking at morally and politically problematic language use from a non-ideal perspective, I point to problems that many existing theories of slurs run into. Thirdly, I propose a specific way of distinguishing between the categories of pejoratives, derogatory terms, slurring terms, and swearwords which is in line with Mihaela Popa-Wyatt’s (2016) observation that slurs (in her use of the term) are especially problematic if they target oppressed groups. Moreover, this categorisation can incorporate Quill Kukla’s (writing as Rebecca Kukla, 2018) very illuminating account of the pragmatic and social functions of slurs and situate their work in a broader picture of morally and politically problematic terms. I end, fourthly, by showing how inferentialism as a content view is well-suited to account for meaning in derogatory language use whilst allowing for further explanations of the communicative functions of slurs.^1^

## Current theories of slurs/derogatory terms

The body of literature on derogatory terms has grown significantly since Christopher Hom’s (2008) overview of the different positions. I outline the main ideas of the existing approaches by distinguishing four types of accounts, marking the most basic differences between them. In contrast to other categorisations that differentiate between semantic and pragmatic views, I group them into content views, hybrid views, misrepresentation views, and linguistically minimalist views.^2^

### Content views

Content views such as *inferentialism* and *thick semantic externalism* hold that the derogatory force of derogatory terms is built into their semantic content. According to these views, the following sentences have different semantic contents:

1. Obama was the first Black person to become president of the US.
2. Obama was the first n·gger to become president of the US.^3^

The basic idea of *inferentialism* is that a sentence’s meaning is determined by the role that sentence plays in the practice of making assertions and giving and asking for reasons, i.e. by the set of sentences that it can be inferred from as well as the set of sentences that can be inferred from it (cf. Brandom, 1998, 2001). The meaning of a term is then determined by the different roles it can play in making assertions. Participants in this practice hold each other accountable to the commitments and entitlements that come with making specific assertions. According to inferentialism, commitments are determined by the conceptual norms of the discursive community. Thus, the meaning of a term is determined by its function in a linguistic practice, rather than the speaker’s intentions.

Since derogatory claims are part of the set of inferences that are to be drawn from the use of a derogatory sentence, inferentialists hold that the derogatory content of a derogatory term is part of its semantics. Asserting (2) commits the speaker to a set of claims including the claim that Black people are inferior to white people. Upon request, the person who uttered (2) would have to agree with the inferiority claim. Otherwise, the audience could rightly accuse her of conceptual confusion.^4^

*Thick semantic externalism*, a truth-conditional version of the content view, is based on the assumption that the meanings of derogatory terms cannot be determined by the mental states of individual speakers alone, but are partly determined by the social institutions and practices that support the use of the derogatory term in question (Hom 2008, 2010; Hom and May 2013, 2018). According to this view, the conditions under which (2) is true depend on a racist ideology and ideology-sustaining social practices with regard to Black people. Focusing on racial epithets, Hom holds that they “express complex, socially constructed, negative properties determined in virtue of standing in the appropriate external, causal connection with racist institutions.” (2008, p. 431) He presents the meaning of such terms in the form of the schematised, complex predicate ‘ought to be subject to x (discriminatory practices) because of having y (negative properties), all because of being z (the term’s so-called neutral correlate).’

### Hybrid views

Hybrid views maintain that the derogatory content does not contribute to the truth-value of the sentence in which the term occurs. According to hybrid views, there is an element of derogatory terms that, even though it does not affect their truth-conditional content, is not cancellable in specific contexts. *Conventional implicature*, *presuppositionalism*, the *embodied gestures account*, and *expressivism* are hybrid views.

*Conventional implicature views* draw on the Gricean framework of implicatures and explain that the derogatory content is conventionally implicated (cf. Potts 2005; Whiting, 2007, 2008, 2013; Williamson, 2009). Take the following sentences as examples:

(3) The Wus are Chinese.
(4) The Wus are ch·nks.

Sentences (3) and (4) do not differ with regard to their truth conditions, but with regard to their conventional content. (4) implies that the Wus are despicable because of being Chinese, or it implies some non-cognitive attitude (Whiting, 2013), while (3) does not. The implication is such that on the one hand, it is detachable, i.e. it applies only to one of the two truth-conditionally equivalent sentences even in the same context. On the other, it is not easily cancellable, so that an utterer of (4) cannot plausibly deny the implicated content (cf. Williamson, 2009, p. 150).

*Presuppositional views* draw on Robert Stalnaker’s notion of a presupposition which can roughly be described as part of the common ground between interlocutors (cf. Cepollaro, 2015; Schlenker, 2007). The presuppositional content “Chinese people are despicable because of being Chinese” is not asserted in (4), but it is taken for granted by speakers and listeners when triggered by the utterance of ‘ch·nk.’ According to Bianca Cepollaro, “the utterance of a slur activates a requirement on the common ground, so that all the participants are taken to share the speaker’s derogatory attitude. In order to avoid that, they need to say something, stop the conversation and prevent the derogatory presupposition from getting into the conversational background” (Cepollaro, 2015, p. 39). Like conventional implicatures, however, presuppositions are not, or not easily, cancellable by the speakers themselves.

Jennifer Hornsby’s *embodied gestures account* models the derogatory content of derogatory terms on the analogy of a bodily gesture accompanying the utterance of the derogatory term’s so-called neutral counterpart.^5^ According to her view, this bodily-gesture-like signal has to be understood in illocutionary terms because it does not contribute to the truth-conditional content itself. Moreover, it is given in the course of speaking, so that something is *done* by uttering the derogatory term. Another characteristic of this gesture-like element is that it does not have a “life of its own, independently of the use of the derogatory word, so that there is nowhere else to look, to appreciate its significance, than to uses of the word” (Hornsby, 2001, p. 140).

*Expressivism* holds that the truth-conditional content of a derogatory term and its so-called neutral counterpart are the same, but by using the derogatory term, speakers express contempt for their targets (cf. Hedger, 2012, 2013; Jeshion, 2013a, b). According to Jeshion, one can hold and express contempt by using a derogatory term without having any “raw” feeling of anger or frustration: “contempt, like resentment, is a highly structured affectively- *and* normatively-guided moral attitude that is subject to evaluation for its appropriateness. As such, in using slurs, speakers not only express their own contempt for the target, but also implicitly represent (but still do not *say* or *assert* that) their targets as *worthy of* contempt” (Jeshion, 2013a, p. 242). Besides the truth-conditional and the expressive component, group-referencing slurs additionally have an identifying component, i.e. by using them, the speaker takes the property ascribed by the derogatory term’s so-called neutral correlate to be a defining feature of the intended target. Someone who uses the term ‘f·ggot,’ for example, takes the target’s homosexuality to be an identifying property.

### Misrepresentation views

Unlike content or hybrid views, defendants of misrepresentation views do not think that slurring or derogatory terms are problematic because their use conveys any specific false content. Rather, *perspectivalism* and *illocutionary misrepresentationalism* point to the problematic modes of thinking which people are engaged in when they use derogatory terms.

*Perspectivalism* holds that those who use derogatory terms signal allegiance to a certain negative perspective on the target group which is absent from their neutral (or a comparatively neutral) counterparts. A perspective has to be understood as an integrated and intuitive, yet open-ended, way of cognising members of a specific group (cf. Camp, 2013, p. 335). A perspective is cognitive, representational and tied to emotions in that it is a tool for thinking and feeling without entailing specific thoughts or being feelings themselves. By using a specific derogatory term and thereby showing their commitment to a certain perspective, the speaker need not be committed to any concrete claim that forms part of the overall perspective.

A different misrepresentation view is what I call *illocutionary misrepresentationalism*. Mark Richard holds that using slurs, which target people based on their social group membership, radically misrepresents these people:

When the anti-Semite thinks of someone in an anti-Semitic way, he thinks in a way that expresses, that vents his negative attitude toward Jews, and thereby shows contempt for and denigrates them. To *do* these things is to misrepresent Jews. It is to misrepresent them not because one is using a word that means something like *contemptible because Jewish*. Rather, it is to misrepresent Jews because one is doing certain things—e.g. expressing negative attitudes and contempt elicited by religion—the doing of which is one way to represent Jews as worthy of contempt. (2008, 26f.)

By using a derogatory term, speakers do not make an assertion, rather they perform a misrepresenting speech act that deprives what is said of truth.

### Linguistically minimalist views

I use the term linguistically minimalist to describe those theories that assign derogatory terms and their neutral counterparts the same linguistic role. According to these views, there is nothing about a derogatory term’s meaning that renders its use problematic. Other social factors prevent us from using derogatory terms. Among these views are *prohibitionism*, the *contrastive choice account,* and *ventriloquistic conversational implicature*.

*Prohibitionism* is the view that derogatory terms are words that a specific discursive community declares to be prohibited, and using them constitutes a violation of this prohibition. Luvell Anderson and Ernie LePore (2013a, b) hold that slurs behave like other taboo words. In doing so Anderson and LePore invert the usual explanation of what makes the use of derogatory terms problematic by claiming that a discursive community first begins to treat a certain word as prohibited. Once this norm of prohibition is set in place, any use of the term in question violates it and causes offense in turn.

According to the *contrastive choice account*, the use of a derogatory term signals that the speaker endorses a set of interrelated derogatory attitudes that are associated with the term. In this regard, the contrastive choice account is similar to perspectivalism. It differs from the latter in that speakers signal this endorsement not simply by their use of a specific derogatory term, but because of their choice not to use the available neutral counterpart. Moreover, this choice is merely a defeasible indicator that the speaker endorses the cluster of negative attitudes. Renée J. Bolinger (2017) contends that the endorsement and, hence, the warranted offensiveness can be cancelled if the speaker does not know about the association between the term and the derogatory attitude. It is also cancellable in cases where the speaker does not know that there is a neutral alternative to the use of the derogatory term and would have used that neutral counterpart had (s)he known about it. Another case of cancelling the warranted offensiveness occurs when no alternative is available or appropriate.

The *ventriloquistic conversational implicature account* draws on the Gricean framework of implicature and his idea of conversational maxims. Whenever interlocutors are taken to follow the maxims, but the conventional meaning of the terms used in their utterance does not suggest so unless the utterance is complemented by an implicated sentence, the missing sentence is conversationally implicated. Just as using the French term ‘scandale’ instead of the English ‘scandal’ to tell the Bill Clinton and Monica Lewinsky story implicates a sexual component that arises from a common American stereotype of the French, ventriloquistic accounts of slurs hold that by using the n-word instead of ‘Black person,’ speakers implicate a certain attitude towards the person they are talking about. According to Nunberg (2018), they use a marked instead of an unmarked term, i.e. they opt out from deploying the expression which is conventionally used in a specific speech community. Whilst conventional implicatures are detachable but not cancellable, conversational implicatures are cancellable but not detachable. They are cancellable because they are implicated according to the conversational maxims of a given speech situation which the participants use to orient themselves. They are not detachable in the sense that they cannot differ between two truth-conditionally equivalent sentences in the same context.

## Non-ideal theorising and the social embeddedness of slurs

Philosophers of language who work on slurring or derogatory terms aim to contribute to an understanding of topical issues within public political discourse. This calls for what feminist and critical race theorists have come to treat as non-ideal theorising. The distinction between ideal and non-ideal theory goes back to John Rawls’s *A Theory of Justice*, and there has been a lot of debate in moral and political philosophy about this distinction ever since. Only recently, the methodological discussions have entered more theoretical areas of philosophy, such as philosophy of language. In reaction to David Beaver and Jason Stanley’s (2019) project of developing the non-ideal philosophy of language, Cappelen and Dever (2021) have challenged the very idea of non-ideal philosophy of language. None of the distinctions that are made between ideal and non-ideal theory in political philosophy, so they argue, can meaningfully be applied to philosophy of language.

However, even if we agree with Cappelen and Dever in that we cannot apply Rawls’s distinction to projects in theoretical philosophy, we do not have to give up on the idea of non-ideal philosophy of language altogether. Several scholars advocate for a methodological understanding of the ideal/non-ideal distinction (Hänel & Müller, 2022; Mills, 2005; Mühlebach, 2022a; Engelhardt & Moran forthcoming; Ernst & Mühlebach forthcoming). If we try to capture what feminist and critical race theorists who explicitly or implicitly commit to non-ideal theorising aim to do, Charles Mills’s (2005) work on ideal theory as ideology proves useful beyond moral and political theory. By drawing on Mills, I have suggested (2022a) that we best understand non-ideal theorising as a commitment to engage in theory-building that does not systematically abstract away from power relations and (social) categories if they crucially shape our object of investigation. Despite disagreements about details of such an approach, this understanding of non-ideal philosophy of language is largely compatible with the recent work by Beaver and Stanley (2019), Jessica Keiser (2022) as well as Engelhardt & Moran (forthcoming).

Slurs and derogatory terms are linguistic phenomena the existence of which heavily draws on power relations. Moreover, there is a broad range of social categories involved in shaping what slurs and derogatory terms are. I thus take it for granted that we philosophers of language who theorise the meaning of slurs and derogatory terms, commit ourselves to attending to these aspects. Note that my understanding of non-ideal theorising does not presuppose a binary of ideal vs. non-ideal theories, but allows for a spectrum: the better your theory captures how power relations and social categories shape your linguistic phenomenon, the more non-ideal it is. The following critical engagement with current theories of slurs is thus best understood as diagnosing shortcomings in living up to that commitment.

What do we want a theory of meaning in derogatory language use to accomplish? I take it that one major aim is to explain the meaning of terms that are morally and politically problematic. I here identify three desiderata for a non-ideal approach to morally and politically significant meaning. First, *use the resources that you have as a political philosopher of language*. Second, *be clear about what makes your object of investigation morally and politically significant.* And third, *choose your object of investigation based on its moral and political significance *(*and thus situate it in a broader landscape of morally and politically significant language*). Most of the available theories in the debate on slurs and derogatory terms fall short with regard to at least one of these desiderata.

I restrict my discussion to these three desiderata without claiming that they are the most important desiderata, let alone the only ones, for any non-ideal theory of slurs or derogatory terms. I discuss these specific problems in an attempt to connect the existing debate on slurs and derogatory terms more with feminist and critical race thinking known for its sensitivity to power relations and the social construction of categories. I thereby hope to help move the debate on slurs and derogatory terms into the direction of opening ourselves more up to criticism that concerns political aspects of slurs and derogatory terms in all their complexity, rather than treating these terms as mainly linguistic phenomena.

### Political philosophy of language and its resources

Slurs and derogatory language use call for a politically informed philosophy of language. Prohibitionists Anderson and Lepore (2013a, b) base their account of slurs on the fact that there are usually strict social constraints that govern the use of such terms. They argue that the offense caused by the use of slurs is a result of violating their prohibition, not of their content or any other linguistic characteristic. Anderson and LePore attempt to do justice to the political part of this phenomenon, but in reversing the direction of explanation, they ignore what philosophy of language can contribute to a better understanding of the phenomenon of derogatory language use. They have been widely criticised on this point. Their answer consists in pointing to a prime example of prohibited words, the tetragrammaton (the Hebrew letters transliterated as *“*YHWH”) in the Jewish religion (see 2013b, 354 ff.). According to them, it is obvious that this name is not prohibited because its content is offensive. Even though they admit that prohibitions are not set in place without reason, they claim that the meaning of the tetragrammaton certainly does not contain any offensive element. They also doubt that there is any reason to believe that the direction of explanation with regard to the tetragrammaton is limited to divine names. Derogatory terms, they insist, are cases in which the prohibition precedes the offensiveness.

Both using the tetragrammaton as an example and attempting to compare this case with derogatory terms are misguided. Firstly, it is unclear whether the example is in fact a prime example for a word that is only offensive because of being tabooed. If we take the religious idea that somebody is partly taking possession of someone if they call them by their name, there might indeed be a reason for not pronouncing the full name of YHWH which is related to its offensiveness. If God is almighty and the most sacred entity, the act of trying to partly take possession of God is offensive, according to a religious view. Hence, the taboo is the consequence of the offensiveness, and not vice versa.

The second and more important concern is that, even if the tetragrammaton were a prime example of originally inoffensive prohibited words, it would still be unclear how it relates to derogatory words. Anderson and LePore doubt that the reason derogatory terms are prohibited lies in their offensiveness, but they do not provide us with any reasons why this should not be the case. Thus, if we are interested not just in knowing *that* but also in understanding *why, in what sense,* and *to what extent* there are strong social constraints on the use of derogatory terms, we ought to make use of some of the tools provided by philosophy of language.

### Moral and political significance of terms

Unlike prohibitionism, all other theories of slurring or derogatory terms use these linguistic tools. They do so by either explaining in what way the use of such terms conveys an attitude of hatred or contempt (Hedger, 2013; Hornsby, 2001; Jeshion, 2013a, b; Nunberg, 2018; Richard, 2008; Whiting, 2013) or why slurring or derogatory terms are offensive (Bolinger, 2017; Camp, 2013; Cepollaro, 2015; Popa-Wyatt, 2016; Popa-Wyatt & Wyatt, 2017; Williamson, 2009).^6^ Robin Jeshion’s expressivism and Renée J. Bolinger’s contrastive choice account provide us with two exemplary positions.

In Jeshion’s case, the wrong-making feature is the expression of contempt, which is “a highly structured affectively- *and* normatively-guided moral attitude that is subject to evaluation for its appropriateness.” (2013a, p. 242) By using a slur, speakers dehumanise and derogate their targets because they express contempt for them. Moreover, Jeshion argues that different slurs all dehumanise their targets to the same degree since the moral attitude does not seem to be gradable.

Bolinger, by contrast, takes offensiveness to be the wrong-making feature of slurs. According to her, if speakers use a slur, they signal their endorsement of a set of interrelated derogatory attitudes which are associated by the term. They do so by choosing not to use the available neutral counterpart. This choice, however, is merely a defeasible indicator that the speaker endorses the cluster of negative attitudes. Endorsement and, hence, the warranted offensiveness can be cancelled if the speaker does not know about the association between the term and the derogatory attitude, if the speaker is not aware that a neutral alternative to the derogatory term exists and would have used this alternative had they known about it, or if the speech act takes place in a situation where an alternative is not available or appropriate.

There are several problems with focusing on the expression of contempt or offensiveness. They cannot account for several characteristics of derogatory terms and thus fail to meet some of the basic criteria with regard to morally and politically problematic language use. Firstly, not all instances of verbal derogation require the speaker to express an attitude of hatred or contempt. Think, for example, of “well-meant” uses of patronising terms such as ‘d·lly bird’. Moreover, the expression of contempt through the use of a derogatory term is much worse if the target is thereby being structurally derogated. For example, the use the n-word has more derogatory force than the use of ‘l·mey’ even if the speaker feels more hatred towards British people than towards Black people.

Secondly, there are numerous cases in which morally unproblematic behaviour causes offense. Certain communities treat public exchange of affection among homosexual couples as offensive, and racist communities whose members believe that reverse racism exists consider certain instances of being called ‘white’ to be offensive.^7^ Offensiveness reflects on the communicative norms of a given society rather than the content of what is done or said. The standard for what counts as offensive in sexist, racist and ableist communities differs significantly from communities with strong anti-sexist, anti-racist, and anti-ableist norms. Lauren Ashwell (2016) rightly holds:

It’s not just that slurring words are impolite, or hurtful, or offensive—these words are objectionable in a way that goes beyond mere offense. Even if no one were in fact offended by the use of a slur, there would still be something wrong with using it. (2016, p. 228)

Stavroula Glezakos’s (2012) discussion of the historical example of ‘Ch·naman’ is useful for this point. Even though this term was not treated as an offensive term for a long time, its common use still contained exoticising and derogatory content all along. With the increasing awareness of this exoticisation and derogation in English speaking communities, this term has come to be considered offensive over time.

Thus, even though both the expression of contempt and offensiveness might be good *prima facie* indicators of morally and politically problematic language use, they are not essential to it. They only become morally and politically significant when paired with structural derogation. As Sally Haslanger observes, many people, “including many philosophers, fail to notice social structures, and when social structures are mentioned, will find the idea mysterious.” (2016, p. 113) However, she rightly points to the fact that some problems are primarily structural and thus call for structural explanations. Structural derogation feeds on social structures that are based on and reinforce social arrangements of oppression. Derogatory practices go far beyond verbal derogation and if we take our speech acts to be embedded in social practices more generally, we should explain in what ways these broader social practices have a bearing on our discursive practices.

For the purposes of this paper, I take structural derogation to maintain and reinforce social structures of oppression. In line with Ann Cudd’s account of structural oppression (2006), oppression often occurs unintentionally, and it even allows for the oppressed to take an active part in their own oppression (Cudd, 1994). A term structurally derogates its target if the norms of its use maintain and reinforce practices which oppress the target’s presumed social group. I speak of structural derogation and derogatory terms rather than oppression and oppressive terms in order to use terms from the literature in political philosophy of language. Whether a term structurally derogates its target is a question of the social function of its use in the first place, rather than the intentions or attitudes of its user.

Our accounts of meaning in morally and politically relevant language use might differ depending on which moral and political theory we have in mind. My own understanding of morally and politically problematic language use draws on the basic assumptions of any relational egalitarian view. This idea contends that human beings have in principle equal authority, status, or standing (cf. Anderson, 2013). Language use is morally and politically problematic if it oppresses people, i.e. if it (partly) constitutes or causes inequality of authority, status, or standing. Even though philosophers of language working on slurs or derogatory terms do not tend to make their commitments with regard to moral theory explicit, it is remarkable that the vast majority of their working examples and the aspects identified as being in need of explanation are in line with this broad moral framework.

### Choice of explananda

With this new focus in mind, let us consider the phenomena that are at the core of currently available theories of slurs and derogatory terms. These theories usually seem to explain the meaning of slurs in general since they do not further specify their object of investigation. However, most theorists base their theories on examples of one specific type of slur: slurs that target people as members of a social group, such as racial slurs (e.g. Anderson & LePore, 2013a, b; Bolinger, 2017; Camp, 2013; Cepollaro, 2015; Hornsby, 2001; Jeshion, 2013a, b; Nunberg, 2018; Richard, 2008; Whiting, 2013; Williamson, 2009). These theories start from the assumption that there is, or in principle could be, an everyday term that serves as a neutral counterpart to the derogatory term under investigation. A neutral counterpart is thought of as a purely descriptive term or expression with the same extension as the derogatory term. Conventional implicature, presupposition, embodied gestures, expressivist, perspectivalist, prohibitionist, and contrastive choice accounts assume that the truth conditions of sentences containing the derogatory term, for example ‘ch·nk,’ are identical to those containing their so-called neutral counterpart, in this case ‘Chinese person.’ Perspectivalism and ventriloquistic conversational implicature do not require the counterpart to be neutral, i.e. purely descriptive. But they still presuppose some everyday language counterpart since the speaker’s choice to use the slur instead of a more standard term explains the offensiveness of the former.

A first problem for all theorists who presuppose neutral counterparts to slurs consists in explaining how their theory can account for other types of slurs. Not all slurs target members of social groups as members of this social group, some call out norm violations by individual members of a social group. Lauren Ashwell has challenged the neutral counterpart assumption with regard to gendered slurs. In her example, ‘sl·t,’ a possible counterpart of the term would be ‘woman who has sex with a lot of partners.’ As Ashwell observes:

Being a woman who has sex with a lot of partners is something that is strongly socially disapproved of, so given the actual social context it is not free of pejorative associations. […] Moreover, this phrase is not purely descriptive. What counts as “a lot of partners” depends on what is thought to be a generally appropriate number of partners. The phrase only means what it means in the context of the particular norms that we have for women’s sexual behaviour, and so is closer in meaning to “woman who has sex with more than an appropriate number of partners for a woman,” or “woman who has more partners than she ought to,” where the number that is said to be appropriate will be fixed by the external social context. (2016, 234f.)

I take philosophers of language to use ‘purely descriptive’ in Ashwell’s first sense—neutral counterparts do not function in any derogatory way. I doubt that defenders of the neutral counterpart assumption are committed to the much stronger claim that neutral counterparts cannot be normative terms at all. This would restrict the set of possible neutral counterparts too much, since many of our social kind terms function normatively.

Ashwell’s critique extends to all derogatory terms which call out the violation of a specific norm rather than targeting people in virtue of being members of a specific social group. Sentences which contain such terms cannot be explained by applying the standard procedure, which consists in firstly identifying the truth conditions with the help of a neutrally descriptive term and then specifying what the derogatory term adds to the picture. I illustrate the difficulties by applying Camp’s perspectivalist account to the two similar terms ‘sl·t’ and ‘wh·re’.^8^

Perspectivalism claims that “by employing a slur a speaker signals a commitment to an overarching perspective on the targeted group as a whole” (2013, p. 337). This perspective is a cognitive tool that structures one’s thoughts so that they hang together in an intuitive whole without any particular thought being necessary or sufficient for the perspective. Applying this view to ‘sl·t’ and ‘wh·re’ leaves us with three options. Firstly, we can identify the social group as the group of women implicated in the speaker’s sexist perspective. There are two reasons why this is unsatisfactory: on the one hand, users of these terms do not apply them to women in general but only to women who exhibit a specific behaviour. On the other, we would need to assume that they are identical in meaning since they differ neither in truth-conditional content nor with regard to the perspective involved. Even if we allow there to be various sexist perspectives, it seems that the perspectives invoked by these two terms do not differ in significant ways. However, these terms do differ in meaning. Whilst both call out an allegedly inappropriate sexual behaviour, only the latter additionally involves a claim about the venality of the target.

The second option consists in assuming that the extensions of ‘sl·t’ and ‘wh·re’ are fixed by ‘woman who is too promiscuous’ and by ‘woman who is too promiscuous and venal’. In this case, the derogatory perspective is already part of the truth-conditional content and we do not have a neutrally descriptive counterpart. Moreover, this option runs counter to Camp’s view that a speaker is not committed to any specific claim which is part of the relevant perspective.

A third option would be to formulate the neutral counterpart by using some meta-description. For ‘sl·t’, for example, we could fix the reference with the expression ‘the kind of woman who has sex with a lot of partners relative to the norms in context C.’ In this case, the user of ‘sl·t’ signals allegiance to the perspective which involves endorsement of the norms of context C. However, here we run into the same problem as before: It commits the speaker to a specific claim, which is not in accordance with Camp’s core assumptions. Furthermore, I doubt that we will be able to describe context C in a fully neutral way. It seems that the choice of our terms to describe C would always either signal our endorsement or show that we are distancing ourselves from the norms of this context.

Even if we were able to give a complicated meta-description, such meta-linguistic or otherwise explanatory expressions are not what philosophers of language have in mind when considering neutral counterparts to derogatory terms. The function of neutral counterparts in these theories is not only to fix the reference, but to determine the basic meaning to which the slur semantically or in most cases pragmatically adds other layers of meaning. Jennifer Hornsby, for example, holds that derogatory terms are useless to us because everything that we would want to do with them can be accomplished by their neutral counterparts (2001, 128ff). Hornsby defends the view that the truth conditions of derogatory terms and their neutral counterparts are identical. Compared to their neutral counterparts, derogatory terms function like pernicious embodied gestures whose significance cannot be fully understood without taking the context of the utterance into account. This is why, according to Hornsby, derogatory terms are useless to “us”: if for every derogatory term that we could use there is another term that does not come with something like a negative bodily gesture, there is no point in using the derogatory one. Whether everything we want to accomplish by using a derogatory term is better accomplished by the use of its neutral counterpart of course depends on who “we” are. But it is clear that the sociologically explanatory expression from above is not useful to anybody in the sense in which the neutral counterpart could easily be used in place of the slur in everyday interactions.

In addition to the difficulties of applying the standard procedure to derogatory or slurring terms for norm violations, there are further complications in finding neutral counterparts to any type of slur. As mentioned above, Glezakos (2012) makes the case for terms such as ‘Ch·naman,’ which was considered to be neutrally descriptive by the vast majority of English speakers but, as historical texts show, functioned in a derogatory way. Her discussion suggests that we need to draw on history, sociology, and psychology in order to see whether a term is derogatory, rather than rely on the speaker’s own assessment.

Moreover, my discussion of semantic contestations (2021) shows that some of the putatively neutral counterpart terms are themselves socially complex to a degree which makes it impossible to take their semantic content to be self-evident. By way of the example of ‘black’, I argue that the semantic content of this politically significant term is contested across different discursive sub-communities, and that some of the contested uses involve verbal derogation. My discussion suggests that the difference between the social kind term use of ‘black’ as ‘racialised-as-black’ and the racialist natural kind term use, which is based on assumed biological differences between races, cannot be captured by pragmatics, but is a point in semantic contestation.

In yet another vein, Jen Foster (forthcoming) compares the pair slur/neutral counterpart to pairs such as ‘chick flick’/‘romantic comedy’ or ‘stoner’/‘cannabis user.’ She argues that these pairs all work similarly in that there is often significant overlap in (presumed) extension and associated stereotypes. This complicates the neat distinction between slurs and neutral counterparts even more.

In light of these complications regarding our object of investigation, it seems that for most theorists, moral and political significance, and as a consequence, oppressive social structures, do not figure most prominently in deciding which phenomena to consider. Racial slurs are among the morally and politically most problematic terms, for sure, but that does not mean that they can be used as the paradigmatic cases for meaning in all kinds of morally and politically problematic language use. If, elaborating on Ashwell’s case of gendered slurs, we fail in applying the truth-conditional standard procedure to a broad range of derogatory or slurring terms, we do not meet the overall aim of illuminating topical discussions on derogatory language use. It is questionable whether accounting for only a small subset of derogatory terms is appropriate. In order to have significant explanatory power, our theories would at least need to explain why the linguistic mechanisms behind the uses of racial and xenophobic slurs differ so much from those of gendered slurs and how our paradigmatic slur cases relate to other pejorative terms such as ‘arsehole’.

Additionally, if neutral counterpart candidates are themselves politically significant terms whose semantic content is sometimes contested, this affects the way in which they can figure as neutral counterparts. Philosophers of language would at least need to specify which use they are taking to be the neutral counterpart use. If Glezakos and myself are correct in claiming that neutral counterpart terms may function in a derogatory manner even though some or even all participants are not aware that their discursive practice is derogatory, then it follows that we need to reconsider the means by which we assess how derogatory specific terms are.

## What to explain? Derogatory content and communicative functions

### A new taxonomy

Given both the narrow scope of many theories of slurring or derogatory terms and the fact that different theories seem to explain different things when talking about such terms, I propose to distinguish between the categories of swearwords, pejoratives, derogatory terms, and slurs. The following is an analytic distinction, and most examples belong to several of these categories. Swearwords, such as ‘fuck,’ violate discursive norms of a given discursive community. Pejoratives more specifically violate such discursive norms by targeting a certain person, a group of persons, or their behaviour. Derogatory terms structurally derogate their targets by virtue of their embeddedness in social structures of oppression. Slurs are those (structurally) derogatory terms which are explicitly so, i.e. they serve a specific communicative function.

Four remarks might be helpful before I go into the details of each category: Firstly, it is a technical distinction. I do not aim to adequately capture the extension of these terms according to ordinary language use but rather provide a taxonomy that allows us to identify discriminatory, i.e. structurally derogating, language use. For example, according to my distinction, ‘whitey’ is a pejorative in most contexts, but not a derogatory term or a slur. This is due to the role of white people in social practices of oppression. Secondly, all of these characteristics come in degrees. The degree of derogation or violation of communicative norms varies among different terms. Thirdly, discursive norms, like any other social norm, change over time. The use of a term may thus violate a discursive norm at a certain point in history, but not at another. Fourthly, this categorisation has considerable overlap with Justina Diaz Legaspe’s (2020) categorisation of slurs, pejoratives, and insults. However, it is more general in at least two respects. First, my categorisation includes terms that have a (structurally) derogatory function but are not (yet) considered to be offensive. It thus embeds slurs and pejoratives with their specific communicative function in a broader landscape of structural derogation and its corresponding language.^9^ Second, according to my characterisation, the label ‘slur’ is not restricted to terms that only apply to groups for which we do have a neutrally descriptive term, i.e. a so-called neutral counterpart to the slur. For example, the category of slurs also includes sexist slurs such as ‘sl·t’ which do not describe a group but call out a norm violation by some members of a social group.

Swearwords have always existed, and they are widely used. As historian Melissa Mohr (2013) shows, swearwords always transgress some specific discursive norms. Contemporary examples of swearwords are ‘fucking’, ‘damn’, and ‘shit’, whereas in the Victorian era, for example, discursive transgressions consisted in using terms such as ‘trousers’ or ‘leg’ since they were too strongly associated with mentioning a person’s genitals. During this time, people used euphemisms such as ‘limb’, which were sometimes even further euphemised, e.g. to ‘lower extremity’ (Mohr, 2013, 192f.). Since discursive norms change slowly but constantly over time, the set of terms which are used for swearing changes significantly over time as well. If speakers violate a discursive norm, they do not necessarily engage in morally and politically problematic language use. Using swearwords may solely consist in venting without harming anybody.

Pejoratives, like swearwords, violate dominant discursive norms, but they do so by targeting people in virtue of some specific behaviour or some perceived group membership.^10^ At first sight, pejoratives all seem to be morally and politically problematic since they target people and violate discursive norms. Their use is offensive and potentially expresses contempt.^11^ However, there are pejoratives which are instances of arguably legitimate social critique. ‘Arsehole,’ for example, calls out the behaviour in which a person takes advantage of their relative position of power. If a friend tells us not to hire arseholes if we want to have a thriving workplace, I suggest that we take this advice seriously and do not hire people who are known for their sexist, racist, or arrogant behaviour, rather than lamenting our friend’s choice of words.

Derogatory terms also target people. In contrast to swearwords, and pejoratives more narrowly, they can but need not be violations of discursive norms. They draw on, promote, and reinforce the structural derogation of the target in question. Against the background of a broadly relational egalitarianist view, the use of derogatory terms is morally and politically problematic because it draws on, is part of, and contributes to broader social practices of oppression. Structural derogation does not need to happen intentionally, nor does it have to be manifest, i.e. acknowledged by any participant in a social practice, in order to be operative. Structural derogation as a moral and political problem provides a reason for treating certain terms as problematic even if most people in a discursive community, or those in the most powerful positions, are not (yet) aware of the derogation. Similarly, it provides us with reasons as to why the use of certain terms, such as ‘whitey’ or ‘arsehole,’ is not a serious instance of morally problematic language even if the targets or maybe even the discursive community as a whole treat it as such.

Slurs are those derogatory terms which explicitly derogate their targets. In cases in which the derogatory nature of certain terms is widely acknowledged, e.g. many racial or gendered derogatory terms, these terms adopt a slurring communicative function. Because their derogatoriness is common knowledge, their use not only structurally derogates their targets, but additionally pragmatically reinforces this derogation. This understanding of slurring terms leaves room for work such as Quill Kukla’s in which they spell out in detail how slurs are mainly to be determined through their pragmatic and social functions that are embedded and reinforce asymmetrical power relations. According to Kukla (2018, 20f.), slurs interpellate, i.e. hail, their targets as being somebody of a certain kind (generic aspect), they ideologically derogate them, i.e. they recognise them as somebody of lesser value, and they exercise power over them by reinforcing the already existing subordination.^12^

I acknowledge that the move of tying the category of slurs to oppressive social structures runs against the widely-held view suggesting that slurs are a general category targeting people by virtue of their membership to a specific group. This widely shared view is certainly a bit closer to our ordinary language use of ‘slur’ than the use suggested by my taxonomy, but it owes us answers to several questions that are not self-evident. For instance, what is the group targeted by gendered slurs? As my discussion above (Sect. 3) suggests, the group in question is not the group of women in general. Hence, what unifies the target group, why is this group a target and what explanatory work does the groupness of it do? Are pejoratives for members of groups such as the Ku-Klux-Klan also slurs? Why (not)? One of the main purposes of this paper is to suggest that adopting a non-ideal perspective means to understand ourselves as implied in politically relevant social practices and structures. If, from a non-ideal perspective, we ask ourselves which discursive phenomena we find important to explain and why, this most certainly moves us to look beyond the categories that our society happens to provide. For instance, my taxonomy suggests that we change the categorisation of ‘whitey’ from being a slur (as it is treated in ordinary language use) to only being potentially pejorative. In my taxonomy it is potentially pejorative because it is potentially violating discursive norms. Changing the categorisation, here, is a sacrifice I am ready to make if my taxonomy helps us get a better sense of what makes certain instances of language use morally and politically problematic.^13^

Given this categorisation of terms, what should philosophers of language focus on? If the goal is to understand meaning in morally and politically problematic language use, we should first pay attention to structural derogation, i.e. its function in maintaining and reinforcing social structures of oppression. A successful theory of structural verbal derogation enables us to explain changes in meaning through changes in social structures and practices over time (e.g. ‘queer’). Moreover, it explains why certain terms are much more pernicious (‘n·gger’) than others (‘l·mey’), and why some instances of offensive speech, such as “speaking truth to power”, are even an appropriate instrument of social critique rather than an instance of morally problematic language use. From a moral point of view, the use of pejoratives is only problematic if it amounts to slurring language use and derogatory terms are always problematic even if their derogatory nature is not yet acknowledged. Furthermore, it allows us to situate straightforwardly derogatory terms in a landscape of politically significant terms and practices more broadly. Finally, structural derogation is crucial for making sense of why people engage in language criticism.

In addition to this work of illuminating structural verbal derogation, philosophers of language are well-equipped to explore the morally relevant pragmatics of slurs specifically, such as their ability to license and incite further, non-verbal violence (Tirrell, 2012) or to actively oppress their targets (Popa-Wyatt, 2016), their various signalling functions (Camp, 2013, Bolinger 2017, Nunberg, 2018), their normalising behaviour function (Diaz Legaspe, 2018), or their reinforcement of in-group and out-group thinking (Tirrell, 2012, 2017). In what follows I shall argue that content views such as thick semantic externalism and especially inferentialism are well-suited to account for meaning in morally and politically significant language use. They both generalise to a broad range of derogatory, swearing, and slurring terms and explain structural derogation while allowing for further pragmatic explanations of the communicative function of slurs.

### Inferentialism as a promising content view of meaning in derogatory language use

Content views in general seem to be in a good position to capture the social embeddedness of derogatory terms in oppressive social structures. In the remainder of this paper, I highlight the prospects of inferentialism as one specific content view by contrasting it to thick semantic externalism.

Thick semantic externalism argues that the derogatory content of derogatory sentences is built into their truth conditions. It does not assume that we can specify the truth-conditional content of any slurring or derogatory term in non-derogatory ways. It thus seems to generalise to a broad range of slurring and otherwise derogatory terms. However, it still presupposes the neutral counterpart assumption. Inferentialism, by contrast, contends that the meaning of a derogatory sentence is determined both by the set of sentences from which it can correctly be inferred and by the set of sentences that can be inferred from its use in a game of making assertions and giving and asking for reasons.^14^ This applies to all assertive sentences regardless of which type of derogatory term they involve. For the inferentialist, there is no need to assume any purely descriptive counterparts to derogatory terms, since everyday language counterparts are only relevant for successful communication, not for semantics. Counterparts to derogatory terms are used whenever a listener needs to communicate that they are able to identify which object the speaker is referring to without endorsing the speaker’s use of the derogatory term.

Participants in the practice of making claims and giving and asking for reasons keep score of the claims that both their interlocutors and they themselves have put forward. They keep track of the commitments and entitlements which, according to the conceptual norms of the discursive community in question, follow the assertions made in this practice.^15^ Scorekeepers are not impartial observers of a game between two other parties, but are directly involved in the practice of making assertions, understanding assertions from other parties and verbally acting upon them. By making an assertion, the speaker is committed to claims that follow materially from her utterance.^16^ She thereby licenses her listener both to ascribe specific commitments to her and to undertake these commitments himself as well. If the listener treats the speaker as being entitled to her assertion, i.e. as saying something true, he thereby commits himself to the assertion and the claims that materially follow from it.

The perspectival character of the social practice of ascribing and undertaking commitments becomes especially salient in the case of derogatory terms. Brandom describes the perspectival character of this practice as follows:

[I]nferential contents are essentially perspectival—they can in principle be specified only from a point of view. What is shared is a capacity to navigate and traverse differences in points of view, to specify contents from different points of view. (1998, p. 485)

The inferentialist’s role is to explain what it is to treat and understand the utterance of the speaker as representing one thing rather than another. With regard to the listener (or scorekeeper) such an explanation entails that she is able to identify which object the speaker is talking about in order to navigate their communication. It neither requires that the listener endorses these inferences, that is, that she acknowledges the inferential commitments herself, nor does it presuppose that any of the specifications, whether made on the part of the speaker or the listener, are neutral in any sense of the term. This accounts for the listener’s ability to grasp the speaker’s meaning without treating the semantic meaning as legitimate.

As an example, take the following assertion:

(5) A n·gger got hired.

If my clearly racist colleague from work asserts (5), there are two ways in which I can restate what my colleague is talking about. I can make a *de dicto* ascription which consists in saying “My colleague says a n·gger got hired.” Or I can make a *de re* ascription by saying “My colleague says *of* a person-racialised-as-black that they got hired.” For our purposes, the difference lies in that only the *de re* ascription allows me both to show that I know who the speaker is talking about and to refrain from the claims she is inferentially committed to by replacing the n-word by another term from my vocabulary. It might seem that making a *de dicto* ascription in which I put the n-word in scare quotes and thus distance myself from the term is a viable alternative to the *de re* ascription. However, it is only a *prima facie* alternative. Using scare quotes in a *de dicto* ascription is not equivalent to deploying a term from my vocabulary in a *de re* ascription, for it does not make explicit what, according to me, my colleague is referring to.

*De re* ascriptions are a handy tool for navigating our communication in those cases in which we do not consider a certain term to be part of our vocabulary. According to my categorisation from above, however, there are various types of seemingly problematic terms and not all of them must be absolutely rejected. And certainly, there are different reasons why people may decide not to use these terms in the case of ‘arsehole,’ ‘f·ggot,’ ‘b·che,’ and the n-word. Whilst the contents of the latter three are worrisome to different degrees, I take ‘arsehole’ to be a helpful term to describe a person, or, sometimes, the actions of a person who arrogantly allows themselves to enjoy special advantages. Under the right circumstances I would both ascribe the commitments that come along with the use of “x is an arsehole” (“x arrogantly allows himself to enjoy special advantages” and “x is worthy of critique”) to the utterer of this claim and undertake these commitments myself as well. The reasons for not using the term in some contexts, then, are not fundamental concerns about this term’s content, but are rather motivated by my wish not to offend somebody too much, or not to foreclose conversation too soon.

Thick semantic externalism explains the force of derogatory terms as a product of invoking an entire ideology together with the discriminatory practices that it supports^17^:

Combinatorial externalism (CE) is the view that racial epithets express complex, socially constructed, negative properties determined in virtue of standing in the appropriate external, causal connection with racist institutions. The meanings of epithets are supported and semantically determined by their corresponding racist institutions. Epithets both insult and threaten their intended targets in deep and specific ways by both predicating negative properties to them and invoking the threat of discriminatory practice towards them. (2008, p. 431)

Hom gives an externalist explanation of derogatory meaning since he takes the content expressed by a derogatory term to be “directly proportional to the turpitude and scope of the supporting racist [or sexist, ableist, etc.] institution that causally supports the epithet” (2008, p. 432). The derogatory force, in turn, is directly proportional to some properties of this externally determined content. Unfortunately, Hom does not say more on why racist webs of beliefs causally lead to socially constructed, (potentially infinitely) complex, negative properties.

Spelling out the relation between problematic institutions and the derogatory content of concepts in inferentialist terms, I contend, explains how the transition from oppressive social practices to the force of derogatory terms works. Moreover, it helps us keep the conceptual norms apart from other social norms. However, in doing so, we shift from talking about the negative properties that are expressed by a term to the pragmatic vocabulary of commitments that a speaker undertakes in uttering a term.

Among people who frequently use the term ‘n·gger,’ for example, social rules are at play which legitimise harmful, derogatory, and stigmatising behaviour against Black people. The conceptual rules guiding the use of the n-word draw on these pernicious social rules. In this highly racist discursive community, the inferences from sentences of the form ‘x is a n·gger’ to ‘x is Black,’ ‘x is despicable,’ and ‘x is inferior to white people’ are all treated as valid inferences. For it is the social practices in which the n-word is used that determine the validity of the inferences. These inferences are not restricted to intra-linguistic transitions from one assertion to another but extend to language-entry and language-exit moves. Certain perceptual circumstances may entitle a speaker to utter an assertion, and specific assertions commit a speaker not only to further claims, but also to specific non-linguistic actions. For example, calling someone in a non-appropriated way a “f·ggot” licenses hostile, rather than loving, behaviour towards the target.

Coming back to Hom’s claim from above, we now have a better grasp of what it means for an underlying pernicious ideology to support and semantically determine the meaning of derogatory terms. Our discursive practices are embedded in our broader social practices. Our conceptual norms are influenced by other social norms in that the latter determine what counts as good evidence or a good reason for something else, or what is epistemically and morally tenable. These broader social practices help constitute a web of inferences from verbally articulated claims to other claims, from perceptual inputs to verbal claims, and from verbal claims to non-verbal actions that are treated as valid. Unlike thick semantic externalism, which assumes that derogatory terms express complex properties that stand in a direct causal relation to pernicious ideologies, the solution proposed here fleshes out the descriptive and derogatory functions of such terms in a pragmatist vocabulary of inferential relations. This brings in social practices right at the beginning of the explanatory enterprise and shows not only *that* ideology and social practices have a semantically relevant function, but also proposes a plausible explanation of *how* they come into play.

The inferentialist framework explains how complex and often ideologically misguided social practices bear on the semantic content of our derogatory terms. In contrast to other views, such as perspectivalism, which also take these broader social practices into account, inferentialism still allows for a fine-grained analysis of conceptual content that captures slight differences in meaning and derogatory force, such as in ‘wh·re’ and ‘sl·t’.

### The inferentialist content view and the communicative function of slurs

According to my categorisation, slurs are derogatory terms whose derogatoriness is explicit, i.e. the dominant discursive norms are such that the use of these terms is negatively sanctioned. Content views of structural verbal derogation and especially inferentialism are in a good position to allow for a broad range of communicative functions of slurs. Since slurs share some of these functions with swearwords and pejoratives, our pragmatic explanations need to be based on a content view of structural derogation if we wish to account for morally and politically problematic uses of language. I will briefly sketch how several explanations of communicative functions of slurs, such as legitimising violent acts, signalling allegiance to a pernicious perspective, and expressing hatred or contempt, relate to an inferentialist view of structural derogation. The list of communicative functions is not meant to be exhaustive.

Lynne Tirrell (2012, 2018) makes the case for the action-engendering function of slurring language use. She discusses the terms ‘iny·nzi’ and ‘inz·ka’ (Eng. ‘cockroach’ and ‘snake’) which were frequently and systematically used in Rwanda’s genocide of the Tutsi at the hands of the Hutu. Tirrell argues that linguistic practices sometimes develop in a way in which they constitute permissibility conditions for pernicious non-verbal actions. Her argument rests on the idea that inferential relations are not restricted to intra-language moves but extend to language-entry and language-exit moves. By changing discursive norms in the time leading up to the genocide of the Tutsi, the inferential connection between ‘inyenzi’ and ‘should be eliminated’ became strong enough to significantly change behavioural norms and expectations. The genocidal practice involved labelling Tutsi as ‘inyenzi’ and thus licensing the act of killing them.

A different function of slurs is that speakers signal their free choice to use the pernicious term, which intensifies the offensiveness of the verbal derogation. This communicative act has been discussed by authors such as Renée J. Bolinger (2017) and Geoffrey Nunberg (2018). They locate the derogatory nature of slurs in the speaker’s choice to use a slur instead of an available counterpart. The inferentialist view differs from their view in two regards. Firstly, as already pointed out, inferentialism takes slurs to be derogatory in virtue of the speaker’s commitments which belong to and reinforce social arrangements of structural derogation of the target in question. Secondly, inferentialism takes this communicative act to rest on the explicitness of a slur’s derogatory nature, not on there being a neutral counterpart. Inferentialists claim that by using a slur, we signal disrespect for the targets and the discursive norms because we and everybody else know that it is derogatory, and we still choose to use it. Whether there is a neutral counterpart to the slur is irrelevant. The slur ‘sl·t’, for example, is explicitly derogatory but there is no everyday neutral counterpart available. Instead of using the slur, we might thus just as well stop judging women with regard to their perceived sexual availability.

A further communicative function of slurs is that by using them, speakers signal allegiance to a pernicious perspective on the target. It makes sense for inferentialists and perspectivalists alike to treat perspectives as open-ended, structured ways of thinking, feeling, and perceiving. Analogously to above, inferentialists take users of slurs to signal allegiance to a pernicious perspective because they choose to use the slur despite its explicit derogatoriness, and not, as Camp (2013) holds, because there is an alternative, neutrally descriptive term available.

As already mentioned, instances of derogatory language can be morally and politically troubling even if the speaker does not express contempt for the target, just as scoring in a particular football game is not necessary for any two teams to have played football. However, we do not fully understand the practice of playing football if we fail to understand that scoring is one of the fundamental aims of this practice. Analogously, the expression of contempt is relevant to derogatory language use in that we do not fully understand the practice of verbally derogating people if we do not take the role of contempt or hatred into account.

## Conclusion

In this paper, I have given an overview of theories of the meaning of slurring and derogatory terms. I have argued that many of the currently available theories face at least one of three problems in explaining meaning in morally and politically significant language use. Morally and politically significant language use calls for non-ideal theorising in the sense that we must not abstract away from power relations and (social) categories that crucially shape the phenomenon we are interested in.

I have first shown that in completely neglecting the linguistic part, as prohibitionists do, they miss out on the opportunity to illuminate how power shapes linguistic meaning as opposed to any other social phenomenon. Such an account ignores the resources that non-ideal philosophy of language provides to illuminate meaning in morally and politically language use. Second, I have argued that theorising how power relations inform morally and politically relevant language use shifts our attention away from offensiveness and expression of hatred towards focusing on language use that contributes to and reinforces oppressive social structures. This focus allows us to make sense of a spectrum between explicitly and only implicitly derogatory terms. With this new focus in mind, I have, third, shown that the assumption of neutral counterparts to derogatory terms is not as innocent as it is commonly presented to be, but rather drastically restricts the scope of explanation. More importantly, it obscures the relationship between derogatory terms that are already acknowledged to be highly pernicious and those derogatory terms that are, often for politically problematic reasons, not yet treated as derogatory by powerful others.

Explaining the specific pragmatics of pejoratives is important, but from a non-ideal perspective, such an explanation serves a different purpose if it is added to the morally and politically problematic content of structurally derogatory terms (‘sl·t’ or ‘ch·nk’) than it does when added to terms that do not contribute to the structural derogation of a person but rather call out their problematic behaviour (‘arsehole’). Philosophers of language committed to non-ideal theorising should be sensitive to such distinctions.

I have suggested that inferentialism as a content view generalises to a broad range of derogatory, swearing, and slurring terms since it does not rely on the assumption that there is a neutrally descriptive counterpart to every derogatory term. I concluded by showing that inferentialism takes both the linguistic and the political aspects of slurs seriously. It is in a good position to explain how structural derogation may enter and shape the semantics of our terms and thus illuminate why and in what way implicitly and explicitly derogatory terms are morally and politically problematic. In addition, the inferentialist view allows us to include a variety of pragmatic explanations that explain, e.g., the communicative function of slurs more specifically.

## Footnotes

1 The aim of this paper is not to provide a full defence or elaboration of an inferentialist account of derogatory terms. I do this elsewhere (2021, 2022b, 2023 and *forthcoming*). For a general inferentialist view see Brandom (1998, 2001), and for a view of derogatory linguistic practices, see also Tirrell (1999, 2012).

2 Among truth-conditional semanticists, there is no consensus as to whether semantics are restricted to truth conditions. Camp (2013), Hedger (2013), and Jeshion (2013a, b), for example, defend semantic accounts that do not identify the derogatory content of a term with its truth-conditional value.

3 I introduce a ‘·’ whenever I do not consider a term to be part of my vocabulary.

4 There are special cases in which the use of the n-word does not commit the speaker to the inferiority claim. I discuss these special cases elsewhere.

5 A neutral counterpart is taken to be a purely descriptive expression with the same extension as the derogatory term in question.

6 Note that Anderson and LePore (2013a, b) also take offensiveness to be the key element of derogatory terms.

7 See, for example: https://jezebel.com/that-white-boy-does-not-like-being-called-that-white-bo-1790749947 (08/18/23).

8 Justina Diaz Legaspe (2018) has recently defended the neutral counterpart assumption against Ashwell’s criticism by distinguishing two types of slur: out grouping vs. normalising slurs. The latter always involve a referential restriction but are thereby still connected to the neutral counterpart. Her discussion does not affect my discussion here since I am not concerned with the question of whether there are in fact neutral counterparts, but only with how several complications with regard to neutral counterparts raise challenges for existing theories of slurring terms.

9 What I call the *communicative function* of slurs and pejoratives here is similar to what Leopold Hess (2022) calls the *expressive meaning* of slurs and pejoratives.

10 This is not our ordinary use of the terms ‘swearwords’ and ‘pejoratives.’ Neither is my use of ‘slurs’ or ‘derogatory terms’ because our ordinary language use does not provide us with any categorisation that I find helpful to systematise morally and politically problematic language use. Here, I want to capture that there are terms that violate discursive norms, and that some of them target people or their behaviour.

11 Violating dominant discursive norms and potentially expressing contempt does not rule out the possibility that many people involved in such practices, including the targets, may find the use of such terms funny.

12 Kukla does not commit themselves to a semantic view of slurs. However, I consider an inferentialist view of slurring terms, according to which semantic content is ultimately determined through pragmatic moves in a game of giving and asking for reasons, to be compatible with their account of slurs as interpellation.

13 Note that I am not suggesting to completely overthrow our ordinary language use of linguistic categories. Nor do I doubt that there might be useful ways to draw on these categories even from a non-ideal perspective. My point is that we should be clear about why the categories which serve as the basis for our politico-linguistic explanations matter to us. And we should make these commitments explicit.

14 This practice does not extend to asserting things such as “he has good handwriting” in the highly-regulated practice of writing letters of recommendation. Even though this is also an assertion, its meaning is being pragmatically shifted because it conversationally implies that the student does not have any other skills. Thus, Hornsby’s concern (2001, p. 138) that the inferentialist model cannot capture how individual speakers use the derogatory term on a particular occasion is misguided. Inferentialists do not hold that particular instances of (derogatory) language use can be explained on the grounds of semantics alone. But if we want to understand why certain words are so suited to be used as weapons while others are not, it is helpful to have a look at their semantics, too.

15 Lynne Tirrell (1999, 146ff.) distinguishes between three kinds of commitments: assertional, referential, and expressive commitments. I think that although the referential and expressive commitments are semantically reducible to basic assertional commitments, distinguishing them will become relevant when we establish a broader inferentialist theory of communication which involves the possibilities of criticising or challenging language use.

16 Brandom’s inferentialism concerns material rules (material inferences, material incompatibilities), and not just formal rules.

17 Hom’s notion of ideology is restricted to a web of beliefs that is complemented by social practices which are supported by the ideology. This conception of ideology is too narrow. Sally Haslanger convincingly suggests that we understand ideology as “the background cognitive and affective frame that gives actions and reactions meaning within a social system and contributes to its survival.” Besides beliefs, this frame includes “more primitive dispositions, habits, conceptual frameworks, and a broader range of attitudes than just belief” (Haslanger 2014, p. 386).
